# The selective aldosterone synthase inhibitor Baxdrostat significantly lowers blood pressure in patients with resistant hypertension

**DOI:** 10.3389/fendo.2022.1097968

**Published:** 2022-12-09

**Authors:** Imma Forzano, Pasquale Mone, Fahimeh Varzideh, Stanislovas S. Jankauskas, Urna Kansakar, Antonio De Luca, Gaetano Santulli

**Affiliations:** ^1^ DDepartment of Medicine, Division of Cardiology, Wilf Family Cardiovascular Research Institute, Einstein-Mount Sinai Diabetes Research Center (ES-DRC), Albert Einstein College of Medicine, New York, NY, United States; ^2^ Department of Mental and Physical Health and Preventive Medicine, Campania University “Luigi Vanvitelli”, Caserta, Italy; ^3^ Department of Molecular Pharmacology, Einstein Institute for Aging Research, Fleischer Institute for Diabetes and Metabolism (FIDAM), Einstein Institute for Neuroimmunology and Inflammation (INI), Albert Einstein College of Medicine, New York, NY, United States

**Keywords:** aldosterone, baxdrostat, blood pressure, BrigHTN, CIN-107, clinical trial, hypertension, resistant hypertension

Resistant hypertension is defined by blood pressure (BP) targets not achieved despite the use of at least 3 anti-hypertensive drugs of different classes, including a diuretic (1). Diagnosed in more than 10% of hypertensive patients, it represents a high-risk phenotype, leading to an increased risk of cardiovascular disease and all-cause mortality (2). A BP that cannot be controlled with the use of at least 5 antihypertensive agents of different classes, including a long-acting thiazide-like diuretic such as chlorthalidone, and spironolactone is defined refractory hypertension. Substantial evidence indicates that aldosterone excess is very common in patients with resistant hypertension and primary aldosteronism is present in ~20% of patients with confirmed resistant hypertension; intriguingly a positive relationship (more pronounced in men) between weight gain and aldosterone levels has also been demonstrated (3, 4). Despite its side effects (5), the mineralocorticoid receptor antagonist spironolactone remains the preferred 4^th^ line add-on therapy in patients with resistant hypertension. The adverse effects of spironolactone (which include reduced testosterone synthesis, hyperkalemia, gynecomastia, breast tenderness, menstrual irregularities and postmenopausal bleeding) are essentially due to the off-target blockade of several steroid hormone receptors (5). To counteract these obstacles, a different approach has been applied, *i.e.* directly targeting the synthesis of aldosterone instead of blocking its receptor. However, Osilodrostat, the first inhibitor of the enzyme aldosterone synthase, was associated with off-target inhibition of cortisol synthesis (6), an effect explained by the >90% sequence similarity between 11β-hydroxylase (the final enzyme required for cortisol synthesis, encoded by the gene CYP11B1) and aldosterone synthase (encoded by the gene CYP11B2) (7).

Baxdrostat, a drug originally developed by Roche (RO6836191) (8) and subsequently licensed to CinCor Pharma, Inc (CIN-107) (9), embodies an exquisite example of selective inhibition of aldosterone synthase, without affecting 11β-hydroxylase. Preclinical studies conducted in cynomolgus monkeys demonstrated that this molecule inhibited aldosterone synthesis without affecting the adrenocorticotropic hormone–induced rise in cortisol (8); these findings were also confirmed in healthy subjects (Clinicaltrials.gov Identifier: NCT01995383) (8). Safety, pharmacokinetics, and pharmacodynamics of multiple ascending doses of Baxdrostat were later tested in a Phase I trial, which confirmed that Baxdrostat was safe and well tolerated and induced a dose-dependent reduction in plasma aldosterone but not on cortisol.

Baxdrostat has been tested in a randomized, double-blind, placebo-controlled, dose-ranging Phase II trial: A Study of CIN-107 in Adults with Treatment-Resistant Hypertension (rHTN) (BrigHTN, Clinicaltrials.gov Identifier: NCT04519658). The results of this clinical trial have been presented by Dr. Mason W. Freeman at the latest Scientific Sessions of the American Heart Association in Chicago (Session “Late-Breaking Science: Resistant Hypertension: A Pressure Cooker”) and simultaneously published in *The New England Journal of Medicine* (10).

The BrigHTN trial was conducted from 30 July 2020 to 14 June 2022, screening 779 individuals, of which 274 were randomly assigned to receive placebo (69 patients), 0.5 mg Baxdrostat (69 patients), 1 mg Baxdrostat (69 patients) or 2 mg Baxdrostat (67 patients). Before randomization, the design of the study included a screening period (up to 8 weeks) and a 2-week run-in period to assess medication adherence (**Figure 1**). At baseline, the main characteristics were similar across all treatment groups. Black patients represented 28% of all participants, 29-46% had diabetes.

**Figure 1:**
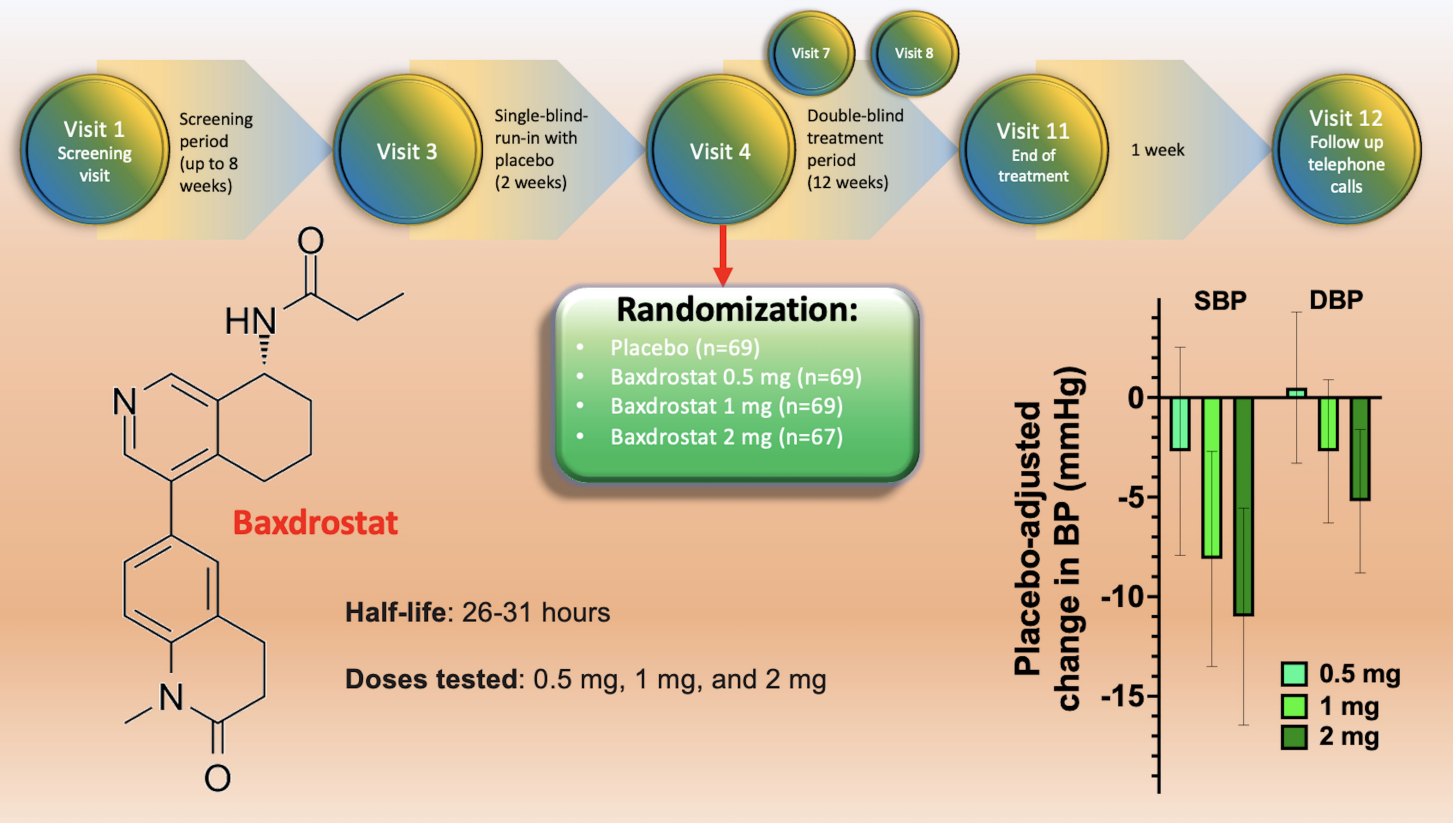
Design of the BrigHTN trial (top), structure of Baxdrostat (right), and representation of the main results in terms of blood pressure reduction at 4 weeks for the three dose groups; SBP, systolic blood pressure; DBP, diastolic blood pressure.

The trial was stopped early for overwhelming efficacy of the drug: indeed, twelve weeks after randomization, Baxdrostat at 1 and 2 mg significantly lowered systolic BP compared to placebo (meeting the primary outcome of the study). The secondary outcome (differences in diastolic BP), was met at the 2 mg dose (10). Exploratory end points included the demonstration that Baxdrostat reached a maximum plasma level in <4h, leading to a dose-dependent decrease in serum aldosterone, without affecting cortisol levels.

In terms of side effects, none of the serious adverse events observed were deemed by the investigators to be related to Baxdrostat. Moreover, none of the patients had to discontinue the trial because of hyperkalemia, which is remarkable: the cases of hyperkalemia observed in a few patients receiving Baxdrostat resolved rapidly with “routine dietary advice” (10); it has to be noted, though, that patients with an estimated glomerular filtration rate >45 ml/min/1.73m^2^, had been excluded. Another noteworthy exclusion criterion, which reduces the generalization of the results of the BrigHTN trial, is having a mean seated systolic BP ≥180 mmHg or diastolic BP ≥110 mmHg. An important limitation of the trial that needs to be highlighted is that the effect of this new drug was only compared to placebo and not to other anti-hypertensive drugs; further investigations in this sense, including phase III trials, are warranted.

In summary, the selective aldosterone synthase inhibitor Baxdrostat leads to significant reduction in both systolic and diastolic BP in patients with resistant hypertension, representing a new powerful tool to treat resistant hypertension.

## Author contributions

All authors listed have made a substantial, direct, and intellectual contribution to the work and approved it for publication.
